# Chemical Composition, Antioxidant Potential, and Blood Glucose Lowering Effect of Aqueous Extract and Essential Oil of *Thymus Serrulatus* Hochst. Ex Benth

**DOI:** 10.3389/fphar.2021.621536

**Published:** 2021-04-29

**Authors:** Tesfay Haile, Susana M. Cardoso, Chirle de Oliveira Raphaelli, Olívia R. Pereira, Elisa dos Santos Pereira, Márcia Vizzotto, Leonardo Nora, Adissu Alemayehu Asfaw, Gomathi Periasamy, Aman Karim

**Affiliations:** ^1^ Department of Pharmacognosy, School of Pharmacy, College of Health Sciences, Mekelle University, Mekelle, Ethiopia; ^2^ LAQV-REQUIMTE, Department of Chemistry, University of Aveiro, Aveiro, Portugal; ^3^ Departamento de Ciência e Tecnologia Agroindustrial, Faculdade de Agronomia Eliseu Maciel, Universidade Federal de Pelotas, Pelotas, Brazil; ^4^ Centro de Investigação de Montanha (CIMO), Instituto Politécnico de Bragança, Campus de Santa Apolónia, Bragança, Portugal; ^5^ Embrapa Clima Temperado, Pelotas, Brazil; ^6^ Department of Pharmaceutical Analysis and Quality Control, School of Pharmacy, College of Health Sciences, Mekelle University, Mekelle, Ethiopia; ^7^ Department of Biological Sciences, National University of Medical Sciences, Rawalpindi, Pakistan

**Keywords:** antioxidant activity, essential oil, thyme-decoction, alpha-amylase, thymus serrulatus, alpha-glucosidase, phenolic compounds profile, antihyperglycemic activity

## Abstract

*Thymus serrulatus,* an endemic plant of Ethiopia, is traditionally used to cure various diseases and as a food ingredient. In the Ethiopian folk medicine, the decoction is orally taken as a remedy to treat diabetes and high blood pressure. The purpose of the present study was to evaluate the antioxidant and antihyperglycemic effects of the aqueous extract and of the essential oil of *Thymus serrulatus*. The chemical composition of the aqueous extract was determined by LC-MS and the essential oil was characterized by GC-MS analysis. Radical scavenging assays, namely scavenging of 2,2-diphenyl-1-picrylhydrazyl (DPPH^•^), hydroxyl (^•^OH), and nitric oxide (^•^NO), were used as a first approach to screen the potential antioxidant abilities of the samples. Alpha-amylase and α-glucosidase inhibitory studies were also employed to evaluate the *in vitro* antihyperglycemic potential of the plant. The *in vivo* blood glucose lowering effect of the extracts was assessed using hypoglycemic activity and the oral glucose tolerance test in normal and in streptozotocin induced diabetic mice. When compared to the aqueous extract, the essential oil showed superior radical scavenging activity, particularly for ^•^NO, as well as greater inhibitory potency against α-amylase and α-glucosidase (IC_50_ = 0.01 mg/ml and 0.11 mg/ml, respectively). Both tested samples showed a statistically significant antihyperglycemic effect. The aqueous extract at 600 mg/kg exerted maximum antihyperglycemic activity (44.14%), followed by the essential oil (30.82%). Body weight and glucose tolerance parameters were also improved by the samples both in normal and diabetic mice. The findings of this study support the hypothesis that aqueous extract and essential oil of *T. serrulatus* are promising therapeutic agents.

## Introduction

Diabetes mellitus (DM), more simply called diabetes, is an endocrine disorder caused by a defect in the pancreas that prevents the production of insulin or its inefficient use by the body, resulting in hyperglycemia that, over time, leads to severe health complications (American Diabetes Association, 2009; Toelsie et al., 2013). DM is a worldwide growing epidemic disorder, with 424.9 million people affected in 2017 and an estimated 48% increase in the number of diabetic individuals by the year 2045 (European Society of Cardiology, 2019).

Chronic hyperglycemic patients live with a high risk of long-term macro- and microvascular complications, such as cardiovascular diseases, nephropathy, retinopathy, and neuropathy (Paul et al., 2020). The ability of free radicals to damage biomolecules is presently accepted to play a key role in late diabetic complications (Asmat et al., 2016). In this context, antioxidant therapy may be considered as one of the important therapeutic strategies in diabetes management (Johansen et al., 2005).

The plant materials being employed as traditional medicines are considered promising sources of new drugs to counteract many diseases, including DM (Doan et al., 2018). In addition to that, plants offer a source of dietary ingredients that affect human physiological functions in order to treat diabetes (Shori, 2015).

The genus *Thymus* (Lamiaceae) includes about 350 species, mostly with a high content of essential oil (Ghasemi Pirbalouti et al., 2015; Salehi et al., 2019). This is mostly distributed in Europe, Asia, and North Africa (Zarshenas and Krenn, 2015) and the vast majority of *Thymus* species are found in and around Mediterranean areas (Niculae et al., 2019). Two native species of thymus, namely *Thymus serrulatus* and *Thymus schimperi*, are found in Ethiopia and both are locally known as *Tosign* (Amharic) and *Tesni/Thasne* (Tigrigna) (Damtie and Mekonnen, 2015).

*Thymus serrulatus* Hochst. Ex Benth is a much-branched perennial subshrub endemic to Ethiopian highlands (2000–4000 m. a.s.l.) of Semien Shoa, Tigray, and Wollo (Damtie and Mekonnen, 2015; Melka et al., 2016). It is restricted to the northern parts of the country and is reported to be found in Alamata and Ofla (Tigray), Yilmana Densa (West Gojjam), and Tarmaber (North Shewa) (Damtie et al., 2017). In turn, *T. serrulatus* is found in different parts of Ethiopia. This specie is commonly used to flavor tea, coffee, and different kinds of stew (Asfaw et al., 2000). Traditionally, the fresh or dried whole parts, leaves, or flowers of the plant are crushed and drunk as a tea to treat different illnesses, including diabetes, high blood pressure, general pain syndrome, abdominal pain, intestinal parasites, and renal disease (Meresa et al., 2017). It is also used for the treatment of headache, earache, and liver disease (Melka et al., 2016). The essential oil of *T. serrulatus* is also used for medicinal purposes as antiseptic, antifungal, and vermifuge properties (Asfaw et al., 2000). The bioactive potential of the plant is supported by previous experimental studies, which include antihelimenthic, antibacterial, fungicidal (Damtie and Mokonen, 2015), diuretic (Melka et al., 2016), vasodilatory (Geleta et al., 2015), and hepatoprotective (Damtie et al., 2017) activity.

Distinct studies previously reported *in vivo*/animal assays with thyme species (Li et al., 2019). In addition, the profile of phenolic compounds (Afonso et al., 2020) and of essential oils (Tohidi et al., 2019) from *Thymus* species originating from different parts of the world have been reported. Still, to our knowledge, there is no report concerning the phytochemical composition and antidiabetic effect of *T. serrulatus*. Therefore, this study evaluated the chemical composition, antioxidant potential, and the antihyperglycemic effects of a decoction and of essential oils from *T. serrulatus* origin.

## Materials and Methods

### Chemicals

The phenolic compounds salvianolic acid B, ferulic acid, apigenin-*O*-glucoside, eriodictyol-7-*O*-glucoside, quercetin-7-*O*-glalactoside, luteolin-7-*O*-glucoside, and rosmarinic acid (obtained from Extrasynthese, Genay Cedex, France) were used as standards for the phenolic characterization of the aqueous extract. Thymol 99% (Sigma Aldrich, Germany) was also used as a standard reference to confirm the identity of the compound in the essential oil. Glibenclamide (Alfa aesar, Great Britain), acarbose (Bayer Pharma AG, Leverkusen, Germany), and quercetin (Sigma- Aldrich, St. Louis, MO, United States) were used as reference standard for the *in vivo* antidiabetic activity, *in vitro* digestive enzyme inhibitory activity, and radical scavenging activity tests of the samples, respectively.

### Plant Materials

The sample of *T. serrulatus* aerial parts was collected during flowering season from Emba Alaje mountain area, Emba Alaje District of South Tigray, Ethiopia (located at 13° 00′ North latitude and 39° 20′East longitude) in the month October 2018. The plant was authenticated by Getinet Masresha (taxonomist) at the Department of Biology, University of Gondar, Ethiopia. A voucher specimen (TH-001/2011) was placed for reference in the University of Gondar’s herbarium.

### Preparation of the Aqueous Extract

The aqueous extract was prepared by decoction, according to Afonso et al. (2017), with adaptations. Aerial parts *T. serrulatus* were shed dried and then powdered. The powdered plant material (20 g) was mixed with distilled water (400 ml) and then boiled for 15 min, left to cool for 5 min, and filtered through Whatman grade 1 filter paper, using a pressurized suction filtration system. Upon concentration *in vacuo* at 40°C, the filtrate was defatted with n-hexane (1:1 v/v). The resulting aqueous extract was freeze-dried, sealed, and kept in a refrigerator for further use. A fresh stock solution was prepared for the experiment whenever required.

### Extraction of the Essential Oil

The fresh aerial parts (250 g) of *T. serrulatus* were cut into small pieces and distilled (hydro-distillation) for 3 h in Clevenger-type apparatus. The process was repeated 11 times to get enough oil. The essential oil obtained was dried with anhydrous sodium sulfate and kept in closed vials at 4°C in a refrigerator (Asfaw et al., 2000). The percent yield of the obtained essential oil was calculated based on the weight of the fresh plant material (*v/w*).

### Phytochemical Characterization of the Aqueous Extract

The phenolic compounds in *T. serrulatus* aqueous extract were identified by UHPLC-DAD-ESI-MS^n^ analysis, performed on a Ultimate 3000 (Dionex Co., Sunnyvale, CA, United States) apparatus equipped with an ultimate 3000 Diode Array Detector (Dionex Co.), and coupled to a mass spectrometer, according to the method previously described by Pereira et al. (2018). The chromatographic apparatus was composed of a quaternary pump, an autosampler, a photodiode-array detector, and an automatic thermostatic column compartment. The column used had a 100 mm length, 2.1 mm i. d., 1.9 µm particle diameter, and end-capped Hypersil Gold C18 column (Thermo Scientific, Waltham, MA, United States), and its temperature was maintained at 30°C. The solvent system used to analyze the sample include water acidified with 0.1% formic acid (*v/v*) (solvent A) and acetonitrile (solvent B). The elution was carried out in binary gradient by gradually increasing the amount of solvent B; from 10–20% (over 6 min), from 20–25% (12 min), from 25–34% (30 min) and increased up to 100% at 37 min maintaining for 3 min, finally returning to the initial conditions at 40 min. The flow rate used was 0.2 ml/min^−1^. The UV–Vis spectral data for all peaks accumulated in the range 200–600 nm and the chromatographic profiles were recorded at 280, 320, and 340 nm. The mass spectrometer used was a Thermo LTQ XL (Thermo Scientific, San Jose, CA, United States) ion trap MS, equipped with an ESI source with Thermo Xcalibur Qual Browser software. The instrument was operated in negative-ion mode and the full scan covered the mass range from *m/z* 100 to 2000. Nitrogen above 99% purity was used and the gas pressure was 520 kPa (75 psi). ESI needle voltage set at 5.00 kV and an ESI capillary temperature of 275°C. CID–MS/MS and MS^n^ experiments were simultaneously acquired for precursor ions using helium as the collision gas with collision energy of 25–35 arbitrary units.

The major compounds in the aqueous extract were quantified using the external standard method by peak integration with the exact or structurally related compounds.

### Phytochemical Analysis of the Essential Oil

The essential oil composition of *T. serrulatus* was analyzed by GC-MS according to the adapted method of Tirillini et al. (2008). For the analysis, a gas chromatograph (Agilent 7820A) equipped with a FID and a capillary column (fused silica DB-5, 30 m × 0.25 mm i. d., 0.25 μm film thickness), coupled with a mass spectrometer (Agilent 5977B) was used. 50 µL of the oil was diluted in 1.5 ml of methanol in order to improve the separation of peaks and the injection volume was 1 µL. The oven conditions were set initially at 60°C and continued for 3 min and then the temperature was increased at 4°C/min to 300°C and held for 15 min. The injector and detector were operated at 250°C, the flow rate of the carrier gas (helium) was adjusted to 1 ml/min and a split ratio of 1:10 was applied. GC-MS system was operated in the EI mode at 70 eV, in the mass range from an *m/z* of 30–500 amu.

The identification of compounds was carried out by matching their mass spectra with mass spectral library of NIST (version 2.2) and the identity of each component was confirmed by comparing their retention indices (generated by Chemstation software) relative to the C6-C22 n-alkanes with those from the literature (Asfaw et al., 2000; Tirillini et al., 2008; Mancini et al., 2015). Thymol was co-eluted as a standard reference to additionally confirm its identity. The percentage composition of the components was obtained by the peak areas normalization method.

### Preparation of Samples for the *In Vitro* Experiments

For *in vitro* assays, the aqueous extract was dissolved in methanol (Sigma-Aldrich®, Milan, Italy) in the concentration of 0.25 mg/ml, after being vortexed (Vortex, Phoenix AP56, Araraquara, São Paulo, Brazil) for 5 min and centrifuged at 4000 rpm at 0°C for 20 min (Eppendorf 5810 R) and the essential oil was diluted in the methanol in the concentration 0.33 mg/ml. The stock solution concentration of quercetin was 2 mg/ml.

### Antioxidant Activity

#### Diphenyl-1-picrylhydrazyl Radical DPPH• Scavenging Activity

The effect of *T. serrulatus* aqueous extract and essential oil against DPPH radical was determined following the method adopted by Vinholes et al. (2017). In this assay, the sample (25 μL) solution (or methanol, in the case of blank) and 0.6 mM DPPH solution (250 μL) were mixed in a 96-well microplate. Quercetin was used as a positive control. After shaking the microplates, the mixture was incubated in dark for 30 min and subsequently measured on a plate reader (Spectra Max 190) at a wavelength of 515 nm. The results were calculated as inhibition percentage (%I).

#### Hydroxyl Radical (^•^OH) Scavenging Activity

The ability of the samples to scavenge the hydroxyl radical was determined according to the method described by Vinholes et al. (2017) with adaptations. Each test sample (25 μL) was reacted by mixing with 110 μL of iron sulfate heptahydrate solution (8 mM, prepared in EDTA-Na 20 μM), 50 μL of hydrogen peroxide solution (7 mM) and 74 μL of salicylic acid solution (3 mM). Quercetin was used as positive control. After shaking, the microplates were incubated at 37°C for 30 min and the absorbance was read at 515 nm. The results were expressed as inhibition percentage (% I).

#### Nitric Oxide Radical (^•^NO) Scavenging Activity

The nitric oxide radical scavenging ability of the *T. serrulatus* aqueous extract and essential oil was determined according to the method described by Vinholes et al. (2011). The samples (50 µL) and sodium nitroprusside (50 μL, 20 mM) were added to a 96-well plate and incubated under the effect of light for 60 min at room temperature. Subsequently, 2% phosphoric acid solution (50 µL) and Griess reagent (50 µL) were added to the mixture and further incubated for 10 min at room temperature in the dark and the absorbance was measured on a micro plate reader (Spectra Max 190), at a wavelength of 562 nm. Quercetin was used as standard. The results were calculated as inhibition percentage (%I).

### *In Vitro* Blood Glucose Lowering Effect

#### Inhibition of α-Amylase Activity

The activity of *T. serrulatus* aqueous extract and essential oil against the α-amylase enzyme was assessed using the procedure described by Satoh et al. (2015) with some adaptations. Each extract (15 μL) was mixed with phosphate buffer (50 μL, pH 7.0) and the α-amylase enzyme (12.5 μL, 241.71 U 62 ml^−1^), followed by incubation at 37°C for 5 min. The soluble starch as a substrate (62.5 μL) was added to initiate the reaction, and further incubated at 37°C for 15 min. The reaction was stopped by adding 1 M hydrochloric acid (12.5 μL) to the reaction mixture. Twenty-five μL of the iodine solution (25 μL, 0.005 M) and potassium iodide (0.005 M) was added for color formation. Acarbose was used as a positive control. The absorbance reading was done on polystyrene microplates containing 96 wells (Spectra Max 190-Molecular Devices) at a wavelength of 690 nm. The α-amylase enzyme inhibitory activity of the samples was expressed as IC_50_ (mg/ml).

#### Inhibition of α-Glucosidase Activity

The inhibition of the α-glucosidase enzyme was determined according to the method described by Vinholes et al. (2011), with some adaptations. The enzymatic samples (10 μL) were added in *p*-nitrophenyl-α-D-glycopyranosidium substrate (50 μL, 3.25 mM) (diluted in pH 7.0 phosphate buffer) solution. The enzyme (50 μL, 9.37 U mL^−1^ diluted in phosphate buffer, pH 7.0) was then added in the mixture to start the reaction followed by incubation of the mixture at 37°C for 10 min. Acarbose was used as a positive control. The absorbance reading was done on polystyrene microplates containing 96 wells in a Spectra Max-Molecular Devices plate reader and was read at a wavelength of 405 nm, immediately after the end of the reaction. The results of inhibitory activity of α-glucosidase enzyme were expressed as IC_50_.

### *In Vivo* Blood Glucose Lowering Effect

#### Preparation of Animals

In this study, healthy albino mice (20–30 g), after random selection, were housed in animal cages each containing groups of six mice with natural night-daytime (12-h light/dark cycle) exposure and at room temperature. The mice were provided standard pellets and tap water *ad libitum*. During the experiments, the mice were fasted overnight (12–14 h) but allowed free access to tap water. The *in vivo* study was approved by the Health Research Ethics Review Committee (ERC1550/2018), College of Health Sciences, Mekelle University and all the procedures involving animals were conducted to the internationally accepted guidelines.

#### Acute Oral Toxicity Test

Acute oral toxicity was performed according to OECD (Organization for Economic Co-operation and Development) guidelines 425. Albino mice of female sex selected by random sampling were fasted for 3–4 h prior to dosing with free access to water. First, 2000 mg/kg dose, dissolved in 2% tween 80, was administered orally to a single mouse for each of the test samples. The mice were then observed for physical or behavioral changes for 24 h with special attention during the first 4 h. Mice were given access to food after 2 h of dosing. After survival of the administered mice for 24 h, four additional mice (for each sample) were administered the same amount of the aqueous extract/essential oil under the same conditions. For any possible toxic effect of the sample, the mice were closely observed in the first 4 h and then at regular intervals up to 14 days.

#### Hypoglycemic Effect on Normoglycemic Mice

In this method, overnight fasted mice of both sexes with normal blood glucose levels (BGL) were divided into eight groups (*n* = 6). The positive control group received standard antidiabetic drug (glibenclamide 10 mg/kg), while the negative control group received the vehicle [2% tween 80 (10 ml/kg)], and the test groups were administered with the aqueous extract of 150 mg/kg, 300 mg/kg, and 600 mg/kg; and essential oil of 150 mg/kg, 300 mg/kg, and 600 mg/kg of *T. serrulatus* as per body weight of the mice.

To assess the hypoglycemic effect of the test substances, whole blood samples from the control and test groups of mice were drawn immediately before (0 h) and after 1, 3, and 6 h following administration of standard drug and test samples, by tail snip method. BGL was measured using a glucometer. Blood samples were taken at least three times and average value was determined. The hypoglycemic effects of the test samples were compared with the BGLs of the control groups (Sah et al., 2011).

#### Oral Glucose Tolerance Test in Normoglycemic Mice

The oral glucose tolerance test was performed according to the procedure described by Lanjhiyana et al. (2011). Overnight fasted mice were divided into nine groups of six mice in each group (*n* = 6). **Group 1** received 10 ml/kg of 2% tween 80 and served as a normal control group and **Group 2** received 2 g/kg of glucose (negative control). **Groups 3, 4, and 5** received aqueous extract of *T. serrulatus* at doses of 150, 300, and 600 mg/kg body weight, and **groups 6, 7, and 8** were given essential oil at doses of 150, 300, and 600 mg/kg body weight. **Group 9** received glibenclamide, 10 mg/kg, and served as a positive control group. Thereafter, a glucose load (2 g/kg body weight) was given to each mouse, except the normal control group, exactly after 30 min post administration of the test samples, standard drug, or vehicle.

Blood glucose profile of each mouse was measured at five time points: immediately prior to dosing (0 min), 30, 60, 90, and 120 min after administration of glucose in order to determine their BGLs. Blood samples from each mouse were drawn from tail vein, using tail snip method, three to four times and the average blood glucose values were taken. The BGLs were measured using a glucometer. The area under the blood glucose concentration curve (AUC) of each group was determined by plotting BGL against a time graph, and it was used to evaluate the overall percentage reduction in post glucose load hyperglycemia.

#### Antihyperglycemic Activity on Streptozotocin Induced Diabetic Mice

Hyperglycemia was induced in overnight fasted mice by injecting streptozotocin (STZ) (55 mg/kg in 0.1 M citrate buffer, pH 4.5) intraperitoneally a single time (Srinivasan and Ramarao, 2007). The changes in BGL and body weight were noted regularly. After three days, mice with fasting blood glucose (FBG) greater than 200 mg/dl were considered diabetic and were used for the antihyperglycemic study.

The antihyperglycemic activity of *T. serrulatus* aqueous extract and essential oil was performed according to the method described by Kabbaoui et al. (2016). The mice were equally divided and randomly assigned into nine groups of six mice (*n* = 6) each: **Group 1**: healthy mice received the vehicle (2% tween 80, 10 ml/kg) and served as normal control group. **Group 2**: diabetic mice received a daily oral dose of 2% tween 80 (10 ml/kg), served as diabetic control group. **Groups 3, 4, and 5**: diabetic mice received a daily oral dose of 150, 300, and 600 mg/kg aqueous extract, respectively. **Groups 6, 7, and 8**: diabetic mice received a daily oral dose of 150, 300, and 600 mg/kg of the essential oil respectively. **Group 9:** diabetic mice received a daily oral dose of 10 mg/kg of the standard drug glibenclamide and served as a positive control group. The test samples, standard drug, and vehicle were started 3 days after induction of diabetes and administered orally on a once daily basis for 21 days using oral gavage.

Antihyperglycemic effect and body weight change were evaluated by estimating the FBG levels and weight on days 0, 7, 14, and 21.

## Statistical Analysis

The results in this study are expressed as mean ± SEM. The *in vitro* antioxidant and blood glucose lowering values were computed using Graph Pad Prism version 8 for Windows. The *in vivo* data were analyzed using SPSS version 25 by means of ANOVA to differentiate between means of all parameters and the source of significant differences was determined by applying Tukey’s post-hoc tests with multiple comparisons. The results were considered significant (*) when *p*-value was less than 0.05.

## Results

### Phenolic Profile of the Aqueous Extract

**FIGURE 1:**
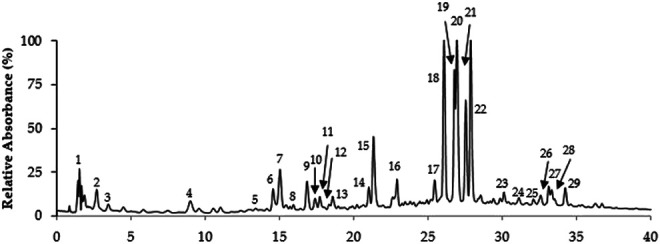
LC-MS Chromatographic profile of *T. serrulatus* aqueous extract.

The extraction yield of *T. serrulatus* aqueous extract was 11.4% (2.28 g). Overall, its LC-MS analysis allowed the identification of distinct phenolic compounds, being this particularly rich in caffeic acid derivatives and salvianolic acids (Figure 1 and Table 1). Among them, the two compounds eluted in peaks 19 and 22 were assigned to two isomers of salvianolic acid K, as they exhibited the main [M-H]^−^ ion at *m/z* 555 in MS spectrum and the major product ions at *m/z* at 493 and 359 in MS^2^ analysis. Notably, these isomers represented almost half (90 mg/g of the extract) of the total quantified phenolic compounds (194.1 ± 15.9 mg/g of the extract). In addition, salvianolic acid B (T_R_ 27.6 min, MW 718), the derivative of salvianolic acid F (T_R_ 15.0 and 16.9 min, MW 376) and salvianolic acid A (T_R_ 34.3 min, MW 494), appeared in moderate amounts. In detail, salvianolic acid B (UV_max_ at 287 and 330 nm) presented *m/z* at 717 and the fragmentation pattern with successive losses of 198 u (danshensu) or 180 u (caffeic acid) units. Salvianolic acid A produced a base peak at *m/z* 295 by the loss of 198 u (danshesu) and other ions characteristics to this salvianolic acid fragmentation.

**TABLE 1:** Phenolic compounds of *T. serrulatus* aqueous extracts determined by UHPLC-DAD-ESI-MS^n^.

NP	T_R_ (min)	λMax (nm)	[H–M]−	MS/MS fragments (*m/z*)	Probable compound	Phenolic content (mg/g)/% composition
1	1.7	206	191	111, 173	Quinic acid	D
2	3.5	280	197	179, 73, 153	Danshensu	6.4 ± 0.9/3.3
3	3.7	279	571	509, 553, 237	Yunnaneic acid E	1.7 ± 0.4/0.88
4	9.0	292, 324	367	193, 191, 173	Feruloylquinic acid	1.3 ± 0.1/0.67
5	13.4	284	387	207, 163, 369	HydroxyJasm acid-*O*-hexoside	D
6	14.6	271, 333	593	473, 503, 353, 383, 575	Apigenin di-*C-*glucoside	5.3 ± 0.4/2.73
7	15.0	290, 324sh	375	313, 269, 179, 135	Salvianolic acid F derivative	12.2 ± 1.7/6.29
8	16.0	283	449	287	Eriodictyol-*O*-hexoside	0.4 ± 0.1/0.21
9	16.9	287, 318sh	375	313, 269, 179, 135	Salvianolic acid F derivative	8.4 ± 0.7/4.33
10	17.4	282, 341	477	301, 299, 277, 191	Quercetin glucuronide	1.9 ± 0.2/0.98
11	17.7	281, 341	463	301	Quercetin-*O*-hexoside	2.1 ± 0.3/1.08
12	18.4	287, 329	553	491, 357, 399, 535	Salvianolic acid C derivative	D
13	18.6	242, 260sh, 341	447	285	Luteolin-7-*O*-glucoside	2.5 ± 0.3/1.29
14	21.1	254, 267sh, 341	593	285	Luteolin-*O*-diglucoside	1.4 ± 0.2/0.72
15	21.3	254, 264sh, 345	461	285	Luteolin-*O*-glucuronide	17.3 ± 1.5/8.91
16	23.0	292sh, 308	521	359, 179, 197, 161	Rosmarinic acid hexoside	2.9 ± 0.2/1.49
17	25.5	287sh, 318	403	367, 385, 359, 313, 223	Rosmarinic acid derivative	2.1 ± 0.3/1.08
18	26.1	290sh, 328	359	161, 179, 197, 223, 133, 123	Rosmarinic acid	19.4 ± 1.5/9.99
19	26.9	287, 326sh	555	493, 359, 401, 537	Salvianolic acid K (isomer 1)	53.8 ± 2.9/27.72
20	27.0	268, 337	461	285	Isoscutellarein*-O*-glucuronide	31.3 ± 3.3/16.13
21	27.6	287, 330sh	717	519, 357, 555, 475	Salvianolic acid B	22.7 ± 1.8/11.7
22	27.9	287, 332sh	555	493, 359, 195, 179	Salvianolic acid K (isomer 2)	37.1 ± 2.7/19.11
23	30.1	252, 266, 344	591	531, 549, 285	Luteolin acetyl dipentoside	1.5 ± 0.2/0.77
24	31.1	287sh, 326	373	179, 161, 135, 197, 329, 355	Methyl rosmarinate	1.3 ± 0.1/0.67
25	32.1	284sh, 340	537	493, 515, 519, 357, 297, 179	Caffeoyl RA (isomer1)	D
26	32.6	287sh, 326	537	493, 515, 359, 375, 357	Caffeoyl RA (isomer2)	1.4 ± 0.1/0.72
27	33.1	284sh, 341	537	493, 519, 515, 357, 339, 179	Caffeoyl RA (isomer3)	D
28	33.4	287sh, 328	537	493, 519, 357, 438, 339, 197	Caffeoyl RA (isomer4)	D
29	34.3	287, 332sh	493	359, 357, 313, 161, 295	Salvianolic acid A	4.7 ± 0.5/2.42
Total quantified phenolic content (mg/g)						194.1 ± 15.9

Values expressed as mg/g of extract; NP, Number of peak; D, Detected; T_R_, Retention time; RA, Rosmarinic acid, sh, shoulder.

Rosmarinic acid (MW 360), a well-known dimer of caffeic acid characteristic from *Thymus* plants, and its derivatives overall represented about 15% of the total phenolics. This compound was eluted in peak 18, which showed the [M− H]^−^ ion at *m/z* 359 and the product ions at *m/z* 161, 179, 197, 223 in MS^2^. Also, two other rosmarinic acid derivatives were found in moderate amounts in the extract. These were eluted in peaks 16 and 17, being characterized by [M− H]^−^ ions at *m/z* 521 and 403, respectively, and the presence of a major product ion at *m/z* 359.

Flavones, namely, isoscutellarein, luteolin, and apigenin glycosides were also representative phenolic constituents of the extract, accounting for 16%, 10% and 3% of the total phenolic content of the extract, respectively. Among these, isoscutellarein-*O*-glucuronide (T_R_ 27.0 min) was assigned due the UV_max_ at 268 and 337 nm and the *m/z* 461→285 consistent with the loss of a glucuronyl moiety (−176 u). In a similar way, the fragment ion at *m/z* 285 and the UV_max_ at 254, 264 and 345 nm, compatible with luteolin, and the main ion [M-H]^−^ at *m/z*, 461 allowed the identification of luteolin-*O*-glucuronide (T_R_ 21.3 min, MW 462), eluted in peak 15. Peak 6 with UV_max_ compatible with apigenin (271, 333 nm) showed a molecular ion at *m/z* 593 and typical *C*-glycosyl fragments at *m/z* 473, 503 and 383, allowing the identification of apigenin di-*C*-glucoside.

### Chemical Composition of the Essential Oil

**FIGURE 2:**
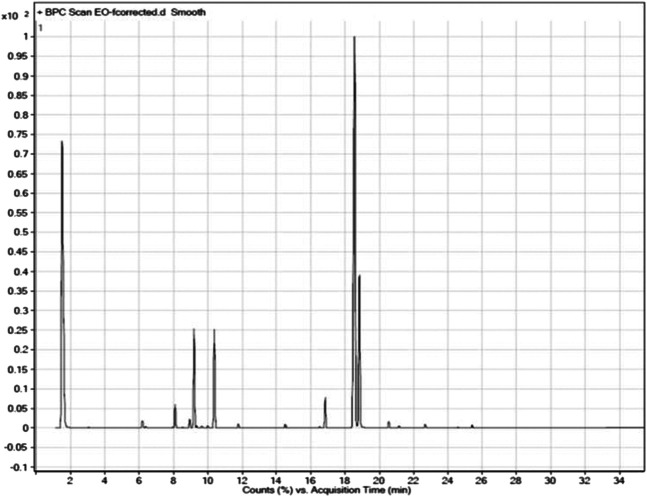
GC-MS chromatographic profile of the essential oil of *T. serrulatus*.

The fresh erial parts of *T. serrulatus* after hydro-distillation yielded 0.9% (24.75 ml) of essential oil, which was characterized by a pale yellowish and a spicy odor. GC-MS analysis of the essential oil revealed 22 components, representing about 99.92% of the total detected constituents. The essential oil was mostly composed of thymol (56.24%), carvacrol (15.44%), *p*-cymene (9.39%), γ-terpinene (9.34%), carvacrol methyl ether (2.77%), and β-myrcene (2.26%). Other components were present in amounts less than 2% (Figure 2).

The composition of the essential oil is summarized in Table 2. For easier comparison and speculating the intended activities of the oil, the components were categorized into five classes of phytochemicals, which were monoterpene hydrocarbons, oxygenated monoterpenes, sesquiterpene hydrocarbons, phenolic compounds, and other components. Accordingly, the essential oil of *T. serrulatus* was mainly composed of phenolic monoterpenes (71.68%) followed by monoterpene hydrocarbons (23.25%).

**TABLE 2:** Chemical composition of the essential oil extracted from the aerial parts of *T. serrulatus*.

Peak N^º^	Compound name	T_R_ (min.)	Ri	% Area	Identification
1	α-Thujene	6.176	931	0.75	1.2
2	α-Pinene	6.374	937	0.14	1.2
3	3-Octanone	7.945	986	0.07	1.2
4	β-Myrcene	8.092	991	2.26	1.2
5	α-Phellandrene	8.531	1003	0.09	1.2
6	α-Terpinene	8.929	1017	0.83	1.2
7	*p*-Cymene	9.201	1022	9.39	1.2
8	d-sylvestrene	9.348	1027	0.15	1.2
9	(Z)-β-ocimene	9.652	1038	0.14	1.2
10	(E)-β-ocimene	9.997	1049	0.17	1.2
11	γ-Terpenene	10.384	1060	9.34	1.2
12	Linalool	11.787	1099	0.35	1.2
13	Terpinen-4-ol	14.520	1177	0.33	1.2
14	Thymol methyl ether	16.520	1235	0.11	1.2
15	Carvacrol methyl ether	16.844	1244	2.77	1.2
16	Thymol	18.572	1291	56.24	1,2,3
17	Carvacrol	18.855	1299	15.44	1.2
18	Thymol acetate	20.540	1355	0.58	1.2
19	Carvacrol acetate	21.147	1371	0.20	1.2
20	(E)-caryophyllene	22.674	1419	0.31	1.2
21	Germacrene D	24.592	1481	0.07	1.2
22	β-Bisabolene	25.409	1509	0.26	1.2

	Total quantified components	**99.92%**	
	Monoterpene hydrocarbons	23.21	
	Oxygenated monoterpenes	4.32	
	Sesquiterpene hydrocarbons	0.64	
	Phenolic compounds	71.68	
	Others	0.07	

**Identification method:** 1, comparison of the Kovats retention indices with published data; 2, comparison of mass spectra with those listed in the NIST 2.2; 3, co-injection with authentic compound.

### *In Vitro* Antioxidant Activity

The antioxidant potential of the essential oil and aqueous extract were determined on the basis of their scavenging activity of the free radicals, namely against DPPH^•^, ^•^OH and ^•^NO. As presented in Table 3, the essential oil and the aqueous extract were both able to eliminate the DPPH and OH radicals, with higher percentages of inhibition for the DPPH^•^. Curiously, essential oil was able to partially scavenge ^•^NO (approximately 35%), while no such ability was registered for the aqueous extract and quercetin.

**TABLE 3:** Antioxidant activity of *Thymus serrulatus* aqueous extract and essential oil.

Sample		Radical capture (%)	
	•DPPH	•OH	•NO
TSEO	94.51 ± 0.08	51.35 ± 0.59	34.89 ± 11.88
TSAE	93.60 ± 0.03	48.89 ± 1.29	ND
Quercetin	92.98 ± 0.38	ND	ND

Results expressed as the mean of % inhibition ± standard deviation. TSEO, *Thymus serrulatus* essential oil; TSAE, *Thymus serrulatus* aqueous extract; ^•^OH, hydroxyl radical; ^**•**^NO, nitric oxide radical; ^**•**^DPPH, 2,2-diphenyl-1-picrylhydrazyl radical; ND, not detected.

### *In Vitro* Blood Glucose Lowering Effect

The essential oil was also more potent than the aqueous extract in regard to the ability to inhibit α-amylase and α-glycosidase enzymes. Also, both the aqueous extract and essential oil were more effective than the drug acarbose against the enzyme α-glycosidase. Moreover, the essential oil was five times more potent than acarbose against the enzyme α-amylase (Table 4).

**TABLE 4:** Inhibitory ability (IC_50,_ mg/mL) of *Thymus serrulatus* aqueous extract and essential oil against the digestive enzymes α-glucosidase and α-amylase.

Sample	IC_50_ value (mg/ml)	
	α-amylase	α-glucosidase
TSEO	0.01 ± 0.00	0.11 ± 0.01
TSAE	24.47 ± 0.29	2.47 ± 0.45
Acarbose	0.05 ± 0.02	16.88 ± 10.69

Results expressed as mean ± standard deviation. TSEO, *Thymus serrulatus* essential oil; TSAE, *Thymus serrulatus* aqueous extract.

### *In Vivo* Blood Glucose Lowering Effect

#### Acute Toxicity Effect

The results of the acute oral toxicity indicated no mortality during the 14 days follow-up. The findings suggested that both the aqueous extract and essential oil are safe as no observable adverse effects were found at the maximum dose 2000 mg/kg body weight.

#### Hypoglycemic Effect

The effects of aqueous extract and essential oil of *T. serrulatus* on FBG level in normoglycemic mice is shown in Table 5. At the lowest dose (150 mg/kg), no significant decrease in blood glucose level was observed in both tested samples. Unlike the essential oil, which did not show any hypoglycemic property at all dose levels, the aqueous extract (at 600 mg/kg) showed statistically significant (*p* < 0.01) reduction in blood glucose level starting from the 3rd h of administration as compared to the negative control. After 6 h of administration, a statistically significant (*p* < 0.001) reduction in blood glucose level was observed at 300 mg/kg of the aqueous extract. Moreover, the effect of 300 mg/kg aqueous extract (22.53%) was comparable with the effect of the standard antidiabetic drug glibenclamide (22.82%) in reducing the blood glucose level. Also, a statistically significant (*p* < 0.001) decrease in the blood glucose level of the positive control group was observed after 1 h of the glibenclamide’s administration.

**TABLE 5:** Hypoglycemic effects of the aqueous extract and essential oil of *T. serrulatus* in normoglycemic mice.

Treatment groups	Fasting blood glucose concentration (mg/dl)				% Change in FBG (hr. 0–6)
	0 h	1 h	3 h	6 h	
1 (negative control)	85.67 ± 0.80	81.17 ± 0.79	75.00 ± 1.13	73.33 ± 0.95	−14.40%
2 (TSAE 150 mg/kg)	85.50 ± 1.20	79.67 ± 0.95	70.33 ± 2.40	77.67 ± 0.92	−9.16%
3 (TSAE 300 mg/kg)	84.33 ± 1.23	84.17 ± 2.52	71.33 ± 2.29	65.33 ± 2.32***	−22.53%
4 (TSAE 600 mg/kg)	76.17 ± 0.87	78.00 ± 0.89	67.83 ± 0.60*	66.67 ± 0.88**	−12.46%
5 (TSEO 150 mg/kg)	75.83 ± 0.31	82.17 ± 0.48	77.00 ± 0.37	81.00 ± 0.63	+6.82%
6 (TSEO 300 mg/kg)	83.83 ± 0.48	84.00 ± 0.45	76.17 ± 0.95	79.33 ± 0.56*	−5.37%
7 (TSEO 600 mg/kg)	77.33 ± 1.02	76.50 ± 1.02	74.17 ± 1.08	75.17 ± 1.05	−2.79%
8 (GB 10 mg/kg)	82.50 ± 0.67	71.00 ± 0.58***	65.83 ± 0.95***	63.67 ± 0.42***	−22.82%

TSAE, aqueous extract of T*. serrulatus*, TSEO: essential oil of T. *serrulatus*, GB, Glibenclamide. Each value is presented as mean ± standard error of the mean (M ± SEM), n = 6, *p < 0.05, **p < 0.01, ***p < 0.001, *, **, *** statistical significance as compared to the negative control group, “+” percent increase in blood glucose level, “−” percent decrease in blood glucose level.

#### Oral Glucose Tolerance Test

**FIGURE 3:**
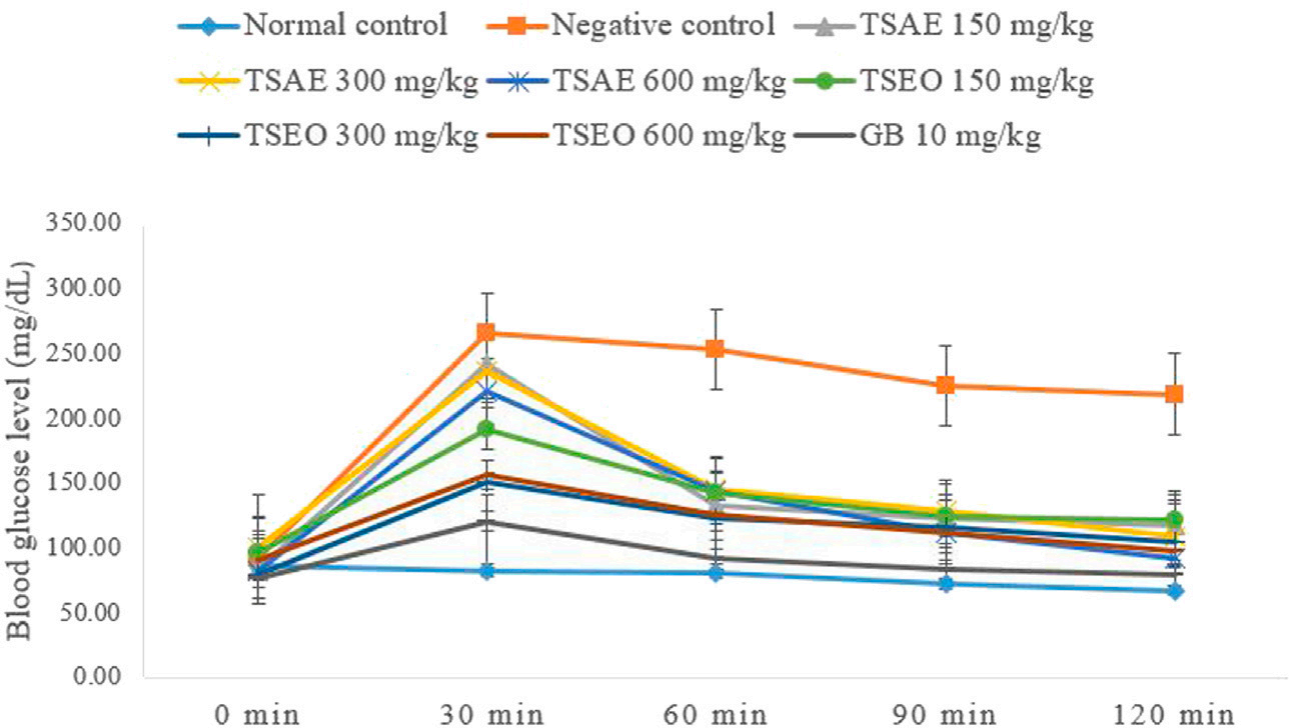
Effects of the aqueous extract and essential oil on OGTT in normoglycemic mice: change in BGL (mg/dl) at different time intervals.

The essential oil and water extract both showed statistically significant (*p* < 0.001) suppression on BGL at all dose levels starting from 30 min of administration of glucose as compared to the negative control group administered with the vehicle (2% tween 80, 10 ml/kg). Except for the normal control, the remaining groups of mice exhibited a peak BGL 30 min after glucose load, which was followed by a decrease over time to the pre-prandial level (Figure 3).

The AUCs of glucose concentration in groups treated with *T. serrulatus* aqueous extract, essential oil, and glibenclamide, were significantly reduced by 36.99% (600 mg/kg), 47.19% (300 mg/kg) and 60.45% (10 mg/kg) respectively, as compared to the negative control group. The essential oil of *T. serrulatus* at all dose levels produced plasma glucose levels significantly lower than those of the aqueous extract at 30, 60, and 120 min after the glucose administration in which the AUC during the OGTT was significantly decreased by 41.35%, 47.19%, and 46.51% at 150, 300, and 600 mg/kg dose levels, respectively.

#### Antihyperglycemic Activity on Streptozotocin-Induced Diabetic Mice

In the three-week treatment course, the fasting blood glucose level of the mice were measured once a week as shown in Table 6. There was no significant difference observed in the fasting blood glucose level between the standard and test groups before initiating the treatment. At the dose of 600 mg/kg, the groups that received standard drug and the test substances showed statistically significant variations in their FBG levels within the first week of treatment (*p* < 0.01 and *p* < 0.001, for the aqueous extract and essential oil respectively). Furthermore, at the end of the second week, the test groups that received 600 mg/kg of both aqueous extract and essential oil (*p* < 0.001) and 300 mg/kg of the essential oil (*p* < 0.05) showed significantly different FBG levels at the end of the second week. On day 21, the test groups that received 150 mg/kg (*p* < 0.05) and 300 mg/kg (*p* < 0.001) of the aqueous extract also showed a statistically significant reduction in their FBG levels.

**TABLE 6:** Effects of aqueous extract and essential oil of *T. serrulatus* on FBG level (mg/dl) in STZ induced diabetic mice.

Treatment groups	Fasting blood glucose level (mg/dl)				% Change in BGL (day 0–21)
	Day 0	Day 7	Day 14	Day 21	
1 (normal control)	97.33 ± 1.15	107.83 ± 1.33	101.67 ± 1.96	91.17 ± 0.95	−6.33%
2 (diabetic control)	271.67 ± 2.73	281.00 ± 5.01	268.17 ± 5.28	270.69 ± 6.42	−0.36%
3 (TSAE 150 mg/kg)	292.83 ± 2.20	291.17 ± 5.13	274.50 ± 4.46	249.83 ± 5.08*	−14.68%
4 (TSAE 300 mg/kg)	284.33 ± 2.09	261.50 ± 4.87	251.83 ± 5.70	228.83 ± 6.82***	−19.52%
5 (TSAE 600 mg/kg)	294.17 ± 2.40	253.00 ± 5.46**	251.83 ± 4.99***	164.33 ± 4.68***	−44.14%
6 (TSEO 150 mg/kg)	281.67 ± 3.13	278.00 ± 6.59	263.83 ± 6.45	254.00 ± 4.82	−9.82%
7 (TSEO 300 mg/kg)	289.00 ± 2.33	274.83 ± 4.30	237.67 ± 12.75*	226.17 ± 5.01***	−21.74%
8 (TSEO 600 mg/kg)	256.83 ± 3.36	243.00 ± 5.98***	194.67 ± 4.19***	177.67 ± 4.33***	−30.82%
9 (GB 10 mg/kg)	288.00 ± 5.08	180.33 ± 4.67***	140.83 ± 6.90***	112.83 ± 5.42***	−60.82%

TSAE, aqueous extract of T. *serrulatus*, TSEO: essential oil of T. *serrulatus*, GB, glibenclamide. Each value is presented as mean ± standard error of the mean (M ± SEM), n = 6, *p < 0.05, **p < 0.01, ***p < 0.001 *, **, *** statistical significance as compared to the negative control group given 2% tween 80 10 mg/kg, “−” percent decrease in blood glucose level.

The aqueous extract and essential oil at the highest dose level (600 mg/kg) exerted maximum antihyperglycemic activity (44.14%) and 30.82% respectively. The extract and essential oil revealed a dose and time dependent antihyperglycemic effect.

#### Effect on Body Weight Changes

In this study, the STZ-induced untreated diabetic group showed 19.97% loss in body weight and the normal control group showed a 4.96% increase during the treatment period. In addition to the diabetic group, the group of mice treated with 150 mg/kg of both of the test substances produced substantial reductions in their body weight as compared to the normal mice. The body weight of diabetic mice receiving glibenclamide and those treated with 600 mg/kg of the aqueous extract and essential oil of *T. serrulatus* showed no significant reduction over the experimental time. A dose dependent improvement in the body weight parameter of mice was observed during the experiment (Table 7).

**TABLE 7:** Effects of aqueous extract and essential oil of *T. serrulatus* on body weight in STZ induced diabetic mice.

Treatment group	Body weight of mice (g)				% Change in body weight
	Day 0	Day 7	Day 14	Day 21	
1 (Normal control)	27.20 ± 1.14	26.73 ± 0.90	27.47 ± 0.91	28.55 ± 1.10	+4.96%
2 (Diabetic control)	24.78 ± 0.81	23.52 ± 0.43	21.80 ± 0.80	19.83 ± 0.42	−19.97%
3 (TSAE 150 mg/kg)	24.82 ± 0.83	23.85 ± 2.07	22.50 ± 0.68	21.08 ± 0.70	−15.05%
4 (TSAE 300 mg/kg)	26.75 ± 1.84	25.22 ± 1.54	24.15 ± 1.89	24.13 ± 0.87	−9.78%
5 (TSAE 600 mg/kg)	24.70 ± 0.61	24.27 ± 0.35	23.78 ± 0.49	24.22 ± 1.10	−1.96%
6 (TSEO 150 mg/kg)	26.12 ± 1.57	24.77 ± 1.92	24.17 ± 1.06	21.30 ± 0.38	−18.44%
7 (TSEO 300 mg/kg)	24.55 ± 1.57	22.55 ± 1.25	22.20 ± 0.58	22.75 ± 0.64	−7.33%
8 (TSEO 600 mg/kg)	23.90 ± 0.38	22.42 ± 0.54	22.92 ± 0.83	23.27 ± 0.66	−2.65%
9 (GB 10 mg/kg)	25.35 ± 1.68	24.45 ± 0.63	24.27 ± 0.39	25.03 ± 1.50	−1.25%

TSAE, aqueous extract of T. serrulatus, TSEO, essential oil of T. serrulatus, GB, Glibenclamide. Each value is presented as mean ± standard error of the mean (M ± SEM), n = 6, “+” percent increase in body weight of mice, “−” percent decrease in body weight of mice.

## Discussion

As the pharmacological activities of different plant extracts are closely associated to their bioactive compounds, the chemical compositions of the aqueous extract and essential oil of *T. serrulatus* were determined. The aqueous extract of *T. serrulatus* was shown to be rich in caffeic acid derivatives (e.g., rosmarinic acid) and salvianolic acids, the latter formed by danshensu linked to caffeic acid moieties (Ma et al., 2019). This finding is in agreement with aqueous decoction of *T. carnosus* in which salvianolic acid K (19.7 mg/g from 42.92 mg/g total phenolics) and salvanolic acid A isomers (16.91 mg/g) constituted the major phenolic content (Martins-Gomes et al., 2018). Even though it is known that *Thymus* species are rich in rosmarinic acid (Pereira and Cardoso, 2013; Afonso et al., 2017), this was clearly less representative (15%) in *T. serrulatus* aqueous extract. These differences in phytochemical contents can depend considerably on extrinsic and intrinsic factors including plant growth, harvest phases, storage, soil, and climatic conditions (Shekarchi et al., 2012). Additionally, isoscutellarein-*O*-glucuronide (16%) and luteolin-*O*-glucuronide (10%) were the most prevalent among the other flavone glycosides. These findings are in agreement with previous reported data that showed *O*-glucuronide derivatives of luteolin and other flavone glycosides have also been detected in different *Thymus* specie*s* in significant amounts (Pereira et al., 2013a; Pereira and Cardoso, 2013; Afonso et al., 2017; Afonso et al., 2018).

The principal constituents of the essential oil were the phenolic monoterpene isomers thymol (56.24%) and carvacrol (15.44%). The findings are in line with a previous study showing that the percent composition of the essential oils of the same plant collected from Ofla and Alamata (South Tigray) were dominated by thymol 49.6 and 65.6% respectively, but the findings differ from those collected from Yilma Densa (West Gojjam, Amhara) that constituted 80.8% carvacrol (Damtie et al., 2018). This suggests that the variations in chemical composition of essential oil can be due to differences in geographic and climatic conditions (Asfaw et al., 2000). The essential oil revealed a high amount of thymol therefore it can be classified as thymol chemotype oil (Tirillini et al., 2008). This finding is in agreement with an earlier study which indicated that the essential oils of *T. serrulatus* belong to the thymol chemotype (Asfaw et al., 2000; Tirillini et al., 2008; Damtie et al., 2017).

Studies show that the increased level of free radicals in the human body is believed to be involved in the pathogenesis of diabetes (Pitocco et al., 2013; Asmat et al., 2016). Thus, the scavenging of these reactive oxygen and nitrogen species is considered to be an effective measure to reduce oxidative stress (Zhang et al., 2015). Thus, phytochemicals with antioxidant properties could play an important role in the prevention and treatment of diabetes (Lin et al., 2016). In this study, both the aqueous extract and the essential oil showed strong radical scavenging activities in the DPPH assay (%I values of 93.60 ± 0.03% and 94.51 ± 0.08%, respectively) as compared to the other assays. Dessalegn and co-researchers also observed strong scavenging effects by various solvent fractions of another thyme specie endemic to Ethiopia (*T. schimperi*) against DPPH radicals (92.2 ± 0.4% to 96.2 ± 1.2%) (Dessalegn et al., 2015).

The free radical scavenging activity of the aqueous extract could be due to the higher content of polyphenolic components like phenolic acids (especially caffeic acid derivatives, mainly salvianolic acids and rosmarinic acid) (Pereira et al., 2013b) and flavones (Catarino et al., 2015). In fact, these classes of phytochemicals have strong antioxidant potential, which are positively correlated with DPPH• scavenging activity (Wang et al., 2019). On the other hand, the antioxidant capacity of the essential oil might be related to its high content of the phenolic monoterpene thymol. This is consistent with the work of Kulisic and coworkers, who reported a high antioxidant activity for the *T. vulgaris* essential oil, which is a thymol chemotype oil (Kulisic et al., 2005). The results of this research support the idea that thymol is a stronger antioxidant than carvacrol, due to the greater steric hindering effects of the phenolic group of the latter (Gedikoğlu et al., 2019). It is known that any imbalance between free radicals and antioxidants leads to the condition known as “oxidative stress” that results in the development of a pathological condition in the body, including diabetes. However, please note that even widely used as a fast and reliable parameter to evaluate the general radical scavenging activity of plant extracts (Ullah et al., 2016), *in vitro* assays lack biological relevance, which must be established through *in vivo* studies.

The anti-hyperglycemic effects of *T. serrulatus* extracts to substantially reduce the elevated levels of blood glucose may be an essential trigger for the development of normal homeostasis during diabetes and its associated complications. Therefore, both *in vitro* (α-amylase and α-glucosidase inhibitory assays) and *in vivo* methods were employed to evaluate the antihyperglycemic potential of the plant.

In the hypoglycemic study, we observed that the aqueous extract, at a dose of 300 mg/kg and 600 mg/kg, significantly lowered BGL compared to negative control in normal mice. Contrary to our results, Shewasinad et al. (2018) reported that the administration of a methanolic leaf extract of *T. serpyllum* to mice caused no significant hypoglycemic effects in mice at all doses. However, the acute oral administration of aqueous extract of *T. serpyllum* leaves (at the dose of 500 mg/kg) showed significant hypoglycemic effect on normoglycemic mice (Alamgeer et al., 2016), which indicates that the effect might be due to the polar phytochemicals present in the aqueous fraction (Issa and Hussen Bule, 2015). Notably, the tested doses of *T. serrulatus* essential oil showed no considerable hypoglycemic effect, suggesting that the mechanism of antidiabetic action of the essential oil might differ from that of the aqueous extract and of the standard drug glibenclamide, which acts by stimulating the pancreatic β-cells to secrete insulin (Tian et al., 1998).

The oral glucose tolerance test, also referred to as the glucose tolerance test, measures the body’s ability to metabolize glucose or clear it out of the bloodstream. The aqueous extract, essential oil, and glibenclamide significantly reduced BGL level in mice by 36.99% (600 mg/kg), 47.19% (300 mg/kg), and 60.45% (10 mg/kg), respectively, as compared to the negative control group. The significant reduction in the BGL registered in the treated groups suggests that both the test substances elicited an increase in glucose utilization and glucose tolerance by the body tissues in mice, which indicates an improved glucose homeostasis (Kabbaoui et al., 2016).

The effect of the relative long-term effect of *T. serrulatus* on BGL was evaluated using STZ-induced diabetic mice. STZ is a diabetogenic agent used in study on rodents (Wu et al., 2015; Radenković et al., 2016) that destructs the pancreatic β-cells, resulting in insufficient insulin secretion. STZ-induced diabetes is reported to resemble the human DM, which is manifested by hyperglycemia, glycosuria, polyphagia, polydipsia, hypercholesterolemia, and body weight loss, which was also observed in the experimentally induced animals in our study (Hammeso et al., 2019).

The aqueous extract at the dose of 600 mg/kg was found to have maximum antihyperglycemic activity (44.14%), followed by the essential oil (30.82%) at the same dose level. This may be explained by the fact that, the test samples are known to exhibit their maximum potential effect at the highest dosage with time against STZ induced diabetes (Rajendiran et al., 2019). Also, these findings are in agreement with the previous reports on antihyperglycemic properties of other *Thymus* species (Benkhayal et al., 2010b; Kabbaoui et al., 2016; Shewasinad et al., 2018). In this sense, the antihyperglycemic properties of the aqueous extract and essential oil of the plant may be related to the presence of high concentration plant secondary metabolites, especially phenolic acids and other polyphenols (in the aqueous extract), and isomeric phenolic monoterpenes thymol and carvacrol in the essential oil (Çam et al., 2017).

Mice with severe hyperglycemia tend to lose a large percentage of their weight after injection of STZ (Ye, et al., 2011). The aqueous extract and essential oil at 600 mg/kg dose level significantly prevented weight loss in mice (−1.96% and −2.65%, respectively) comparable to glibenclamide (−1.25%). Similar findings were also observed on the aqueous leaf extract of *T. satureioides* at 500 mg/kg body weight (Kabbaoui et al., 2016). Some studies suggest that increase in body weight could be attributed to the protective effect of the plant extract against degradation of structural proteins, lipids, and muscle wasting, possibly due to improvement of glycemic control via enhancement of insulin secretion or/and action (Kumar et al., 2014; Emiru et al., 2020).

Regarding the *in vitro* carbohydrate digestive enzyme inhibitory studies, the essential oil showed potent α-amylase inhibitory activity (IC_50_ = 0.01 ± 0.00 mg/ml) as compared to the antidiabetic drug acarbose (IC_50_ = 0.05 ± 0.02 mg/ml) and the aqueous extract (IC_50_ = 24.47 ± 0.29 mg/ml). Likewise, the essential oil also demonstrated the highest α-glucosidase inhibitory (IC_50_ = 0.11 ± 0.01 mg/ml) activity. Previous studies showed a similar trend in terms of α-glucosidase inhibitory activity of *T. albicans*, *T. carnosus* and *T. mastichina* essential oils (Aazza et al., 2016). On the other hand, the aqueous extract revealed better inhibitory activity against α-glucosidase (about 10 times greater) than the α-amylase inhibitory activity. Therefore, the antihyperglycemic effect of the aqueous extract could be partly associated with its ability to inhibit α-glucosidase, which delays the postprandial rise in blood glucose by retarding the digestion of carbohydrate (Al-Hajj et al., 2016). A more effective digestive enzyme inhibitor is expected when it shows mild inhibitory activity for α-amylase but strongly inhibit α-glucosidase enzyme as intestinal disorders such as diarrhea, abdominal pain, and flatulence occur as result of complete inhibition of α-amylase enzyme. This occurs because the starch undigested by the enzyme is used by the intestinal microflora, resulting in gas formation (Cho et al., 2011). These are important findings open up a possibility of the hypoglycemic activity of *T. serrulatus* to control postprandial hyperglycemia via the inhibition carbohydrate digestive enzymes that offer an attractive strategy by delaying glucose absorption and which can potentially slow down the progression of diabetes.

It is argued that postprandial plasma glucose level is more reflective of glucose control than fasting plasma glucose, thus this may be more useful for monitoring diabetic patients (Shahram and Nargess, 2008; Min Park et al., 2009; Lim et al., 2017). The elevated postprandial glucose level is considered an independent risk factor for the development of macrovascular complications associated with diabetes and impaired glucose tolerance. It is also been linked with cardiovascular complications despite the HbA1c values being in the non-diabetic range (Aravind et al., 2015).

Oxidative stress results from an increased level of reactive oxygen species in the body, which are believed to be involved in the pathogenesis of diabetes (Kaneto et al., 2010). Hence, phytochemicals with antioxidant activity can play a significant role in the treatment of diabetes related disorders (Catarino et al., 2015). Another approach to treat hyperglycemic condition is to use drugs that lower the glucose absorption by competitively inhibiting intestinal carbohydrate hydrolyzing enzymes and to decrease inflammatory conditions involved in the pathology of type 2 diabetes (Ota and Ulrih, 2017). Further, the phytochemicals present in the plant may also have the properties to increase secretion of insulin by stimulating or regenerating the β-cells of islets of Langerhans, which may finally improve the activity of carbohydrate metabolizing enzymes toward the re-establishment of normal BGL (Oh, 2015). For instance, phenolic acids like rosmarinic acid, one of the main components of the aqueous extract, is proven to enhance glucose utilization and insulin sensitivity beyond its antioxidant activity (Runtuwene et al., 2016). The antidiabetic prospective associated with flavones including apigenin and luteolin glycosides are known to enhance insulin action, augment the expression and transcriptional activation of PPARγ (peroxisome proliferator-activated receptor gamma) target genes, reduce oxidative stress and inflammation in muscle and promote translocation of GLUT4 (Hossain et al., 2016). Although the antidiabetic effect of thymol and its underlying mechanism of action needs to be furthered explored, it has potential as an anti-hyperglycemic agent by improving insulin resistance in diabetic mice (Saravanan and Pari, 2015).

Hyperglycemia induced oxidative stress via mitochondrial electron-transport chain and subsequent activation of the inflammatory response are considered to be major contributor to diabetes related disorders (Santilli et al., 2015). Therefore, phenolic compounds such as phenolic acids and flavonoids, as antioxidants, have a beneficial role in preventing and improving vascular diabetic complications (Aryaeian et al., 2017) and enhance diabetic wound healing process (Thent and Latiff, 2017). Phytochemicals with antioxidant activity were also proven to revert atherosclerotic cardiovascular complications such as coronary artery diseases by reducing the elevated serum lipid levels in diabetic patients (Bacanli et al., 2019; Hirano, 2018).

## Conclusion

In conclusion, this study suggested that both the aqueous extract and essential oil of *T. serrulatus* have potential antioxidant scavenging ability. Moreover, the two *T. serrulatus* samples hold potency to inhibit α-amylase and α-glucosidase enzymes, which are key metabolic enzymes and targets for diabetes treatment. In addition, the results also demonstrated that *T. serrulatus* was capable of inducing significant antihyperglycemic activities in normoglycemic and STZ induced diabetic mice, as well as of effectively preventing the anticipated body weight loss of the diabetic mice. The overall results suggest that the presence of phenolic constituents in the test samples increased the blood glucose lowering effects of *T. serrulatus*, although further analysis using specific metabolites of these compounds must be tested in order to consolidate such theory. Note also that although the extract and essential oil showed strong radical scavenging activity in different chemical assays, their effectiveness still needs to be evaluated in animal models. We also recommend further investigation to evaluate the anti-diabetic effect of the plant extract and essential oil at lower dose levels to translate the effect closer to a therapeutic value for human studies.

## Data Availability

The raw data supporting the conclusions of this article will be made available by the authors, without undue reservation.
